# Supplementary material for: Flexible adaptation of task-positive brain networks predicts efficiency of evidence accumulation
Source: Commun Biol. 2024 Jul 2;7:801. doi: 10.1038/s42003-024-06506-w (PMC11220037; doi:10.1038/s42003-024-06506-w)
Supplement: Supplementary file 2 — Supplemental Materials [file 42003_2024_6506_MOESM2_ESM.pdf]

Supplemental Materials for:

Flexible adaptation of task-positive brain networks predicts efficiency of evidence accumulation

Alexander Weigard[1], Mike Angstadt[1], Aman Taxali[1], Andrew Heathcote[2] Mary Heitzeg[1], & Chandra Sripada[1]

[1]Department of Psychiatry, University of Michigan

[2]Department of Psychological Methods, University of Amsterdam, Netherlands

Diffusion Decision Model Fit and Prior Sensitivity

Posterior predictive plots of the diffusion decision model (DDM) description of the Human Connectome Project (HCP) and Adolescent Brain Cognitive Development Study (ABCD) n-back task data are displayed in Supplemental Figures 1-2 below. The DDM provided an excellent description of accuracy rates across all conditions and generally provided an accurate description of response time latency, although it slightly overestimated several longer response time quantiles for target trials. Overall, the DDM provides an adequate description of trends in the choice response time data in both load conditions and in both samples.

To assess the potential impact of prior settings on our inferences, we evaluated correlations between estimates of the main parameter of interest (EEA, indexed as the mean of all drift rate parameters) that were estimated under the informative priors we used for each data set (the ABCD-derived priors for ABCD and the same priors with scales multiplied by 1.5 for HCP) and much broader and uninformative priors (Supplemental Table 1). In both samples, EEA estimates were almost perfectly correlated (ABCD $r = 0.97$; HCP $r = 0.99$) between the two different sets of priors. Hence, we can be confident that prior choice has a negligible impact on inferences drawn from the study.

Sensitivity Analyses Without Covariate Correction

We conducted sensitivity analyses for all primary analyses involving mean network activation measures without adjusting values for covariates in order to gauge the potential impact of our covariate controls on substantive inferences. Comparison of values in Supplemental Table 1 from these unadjusted analyses with those in Table 1 of the main manuscript indicates that, although most relationships between network mean measures and efficiency of evidence accumulation (EEA) appear to be slightly stronger without covariate adjustment, the general pattern of these relationships is nearly identical. Correlations between 0-back and 2-back activations in task-positive networks without covariate adjustment were also practically identical to those reported in the main paper for both HCP (FPN $r = 0.64$, CI = 0.60 – 0.68; DAN $r = 0.74$, CI = 0.70 – 0.76) and ABCD (FPN $r = 0.24$, CI = 0.21 – 0.27; DAN $r = 0.35$, CI = 0.33 – 0.38). The dynamic relations between 0-back activation, 2-back activation and EEA also remained the same (Supplemental Figure 4). Taken together, these sensitivity analyses indicate that covariate corrections used in the main manuscript had little impact on the main substantive findings of the study.

Supplemental Figure 1. Posterior predictive plots for diffusion decision model (DDM) fits to the Human Connectome Project (HCP) n-back task data in each cognitive load and trial condition. Plots display the cumulative probability of a "non-target" (solid line) and "target" (dotted line) response for empirical (thick line) and model-predicted (thin line) data. Specific response time (RT) quantiles (.1, .3, .5, .7, .9) are also displayed for empirical (open dots) and model-predicted (solid dots) data.

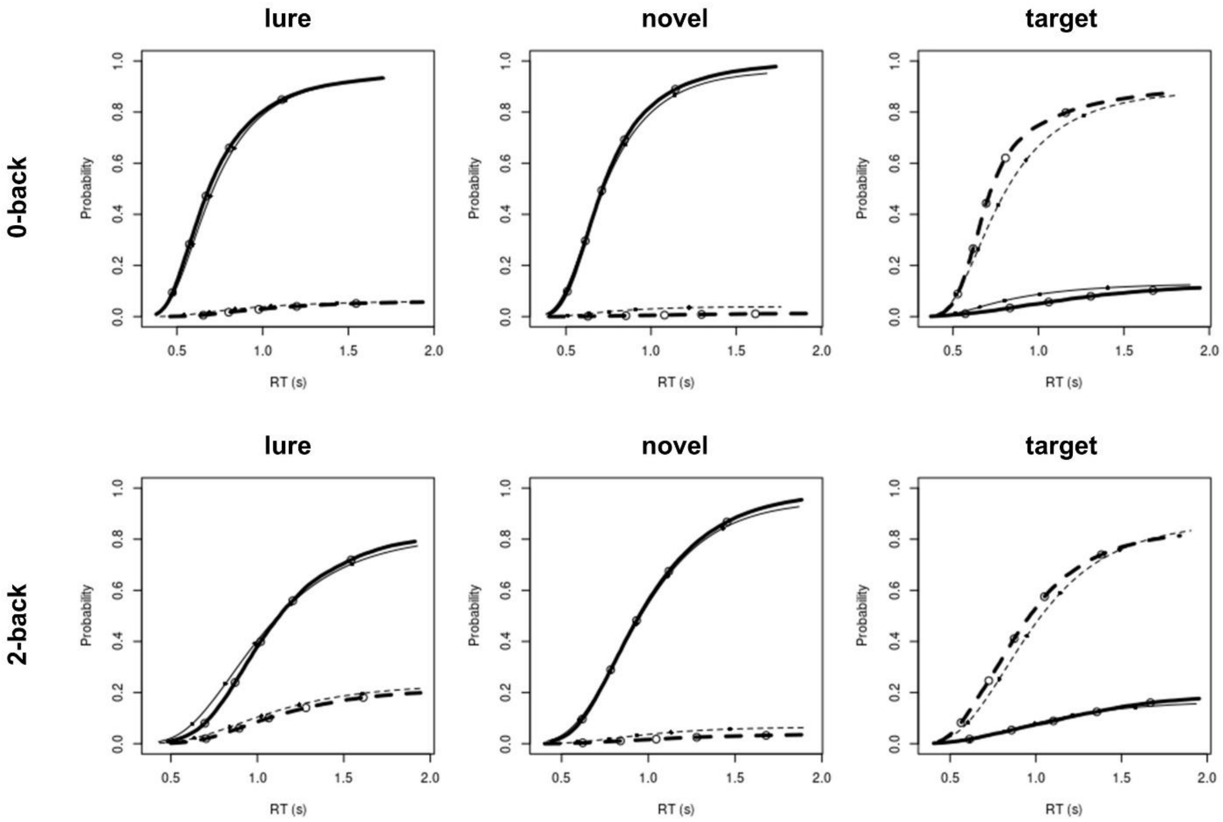

Supplemental Figure 2. Posterior predictive plots for diffusion decision model (DDM) fits to the Adolescent Brain Cognitive Development Study (ABCD) n-back task data in each cognitive load and trial condition. Plots display the cumulative probability of a "non-target" (solid line) and "target" (dotted line) response for empirical (thick line) and model-predicted (thin line) data. Specific response time (RT) quantiles (.1, .3, .5, .7, .9) are also displayed for empirical (open dots) and model-predicted (solid dots) data.

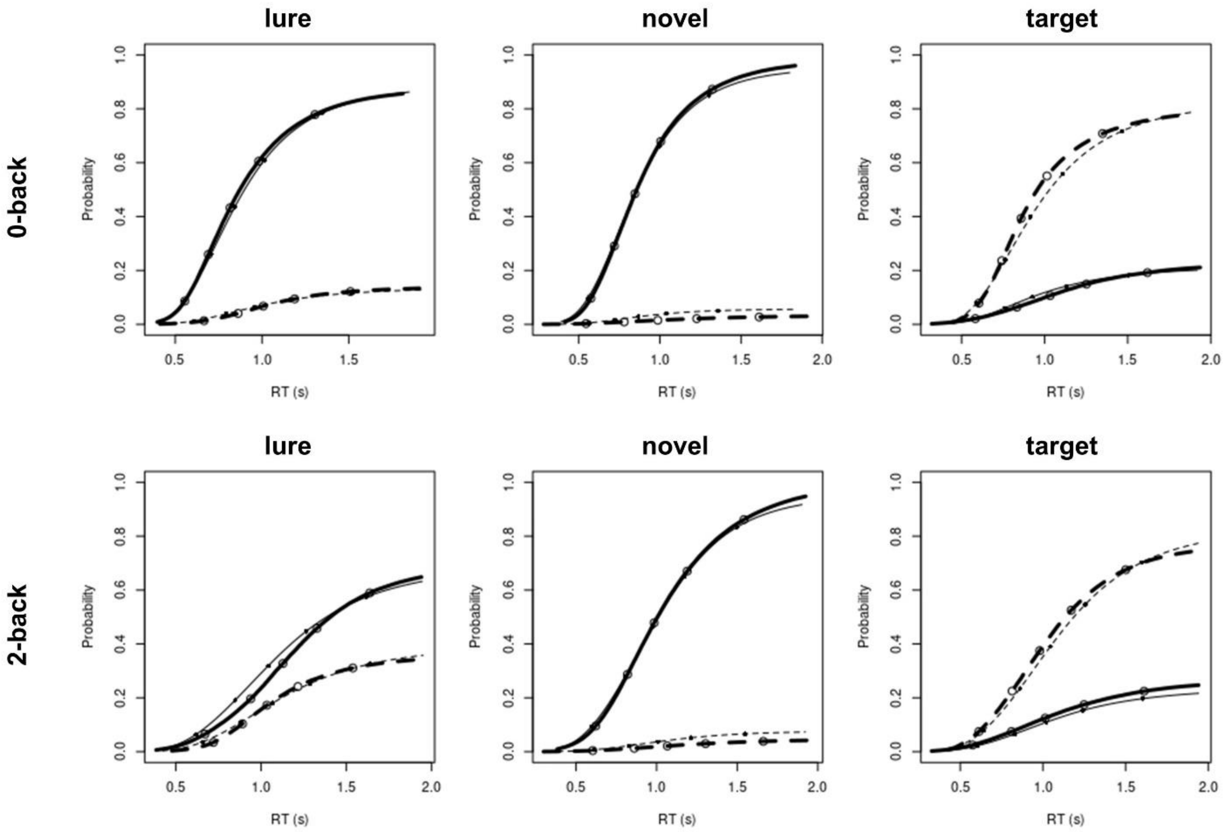

Supplemental Figure 3. Visualization of the steps involved in the multivariate predictive modeling method, cross-validated principal components regression (PCR), that is used in the study.

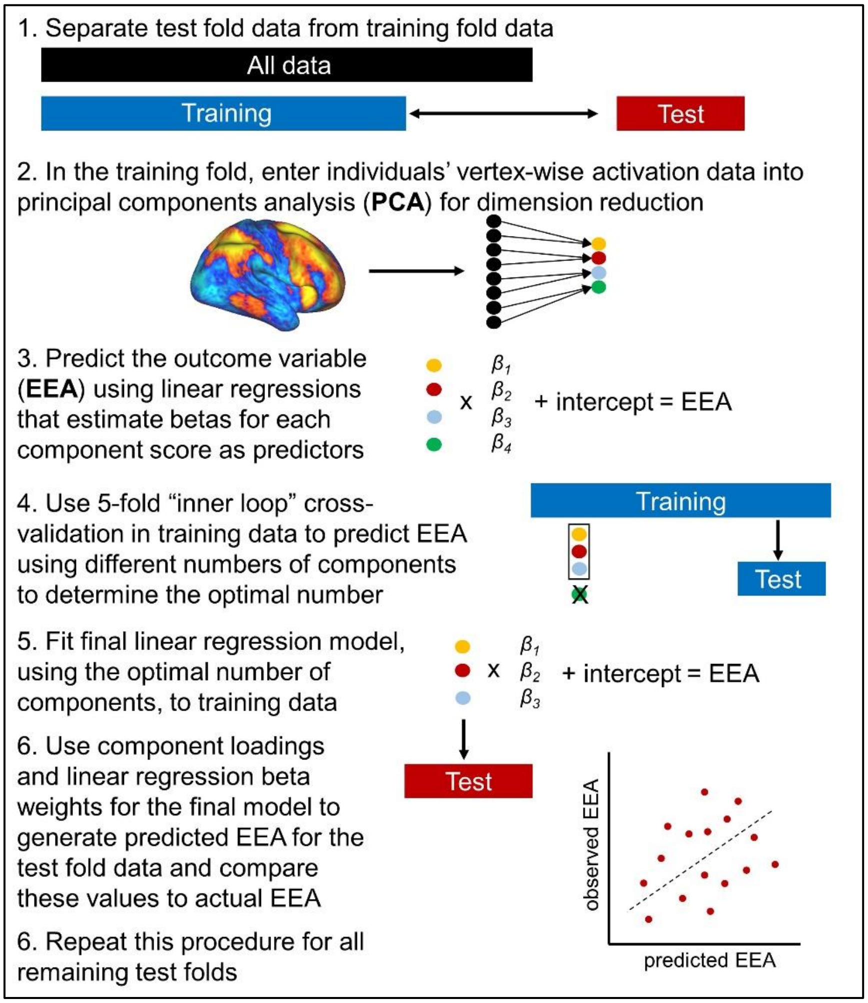

Supplemental Figure 4. Visualization of dynamic relations between average task-positive network activation in the 0-back and 2-back conditions and overall efficiency of evidence accumulation (EEA) on the task for the Adolescent Brain Cognitive Development Study (ABCD; left column) and Human Connectome Project (HCP; right column) samples without adjustments for covariates. Values were converted to standardized scores (Z-scores: mean = 0, SD = 1) for interpretability. Individuals' EEA is represented by the hue of the points, with individuals higher in EEA having darker red hues. Activations of the frontoparietal network (FPN) are shown in the top row while activations of the dorsal attention network (DAN) are shown in the bottom row. Black dotted lines represent the regression line for relations between 0-back and 2-back task activations. Combined with the gray dotted lines representing the average 0-back activation level, the regression lines form four quadrants that denote whether individuals have higher or lower 2-back activation than would be expected given their level of 0-back activation. Bold numbers reflect the average EEA of individuals in each quadrant.

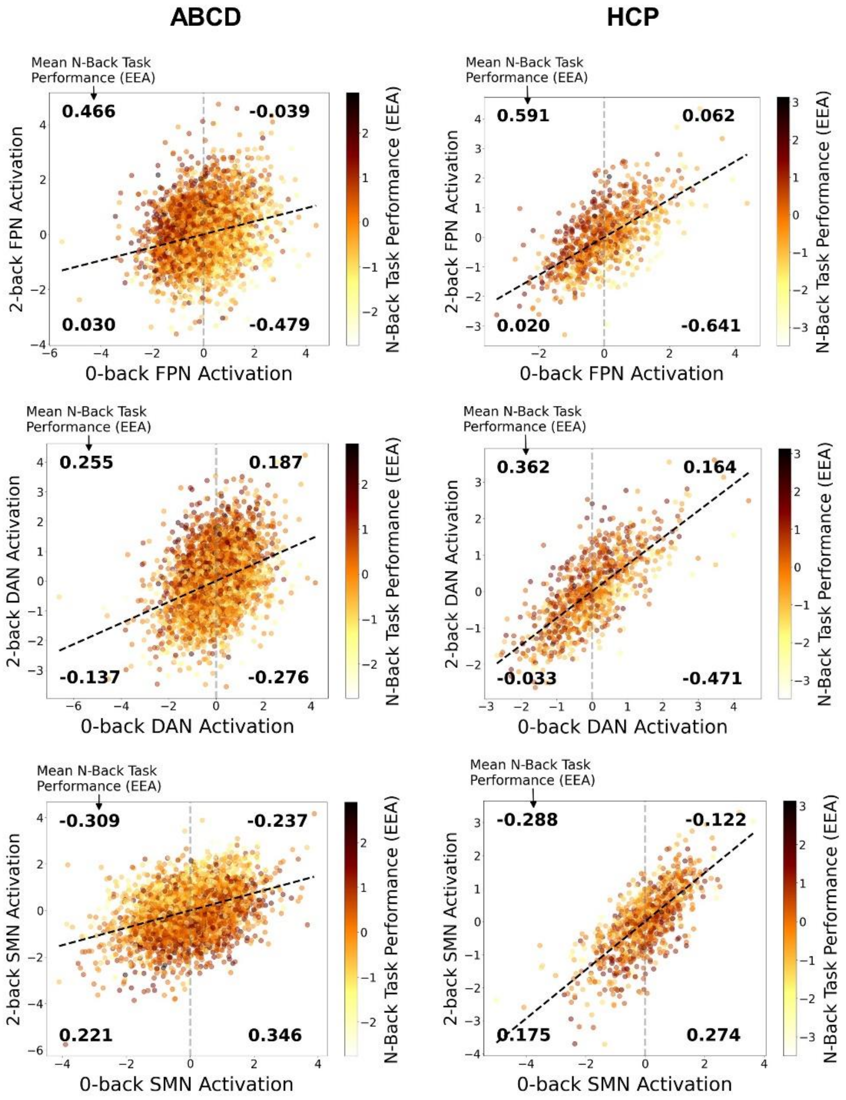

Supplemental Table 1. Priors for diffusion model parameters across both n-back conditions, including the broad, uninformative priors used in sensitivity analyses and the ABCD-derived priors that were used to inform parameter estimation for the main study analyses. All priors were truncated normal distributions defined by mean (μ) and scale (σ) parameters and bounded at the reported values.

			uninformative priors		ABCD-derived priors	
n-back condition	parameter	bounds	μ	σ	μ	σ
0-back	a	0, ∞	1.000	0.500	1.582	0.143
	v_{lure}	-∞, ∞	3.000	1.000	1.246	0.918
	v_{novel}	-∞, ∞	3.000	1.000	1.750	0.409
	v_{target}	-∞, ∞	3.000	1.000	0.961	0.579
	z	0, 1	0.500	0.100	0.487	0.050
	$t0$	0, 2	0.300	0.100	0.310	0.107
	$st0$	0, 2	0.100	0.050	0.431	0.183
	p_{gf}	-∞, ∞	0.000	1.000	-2.343	0.691
2-back	a	0, ∞	1.000	0.500	1.650	0.138
	v_{lure}	-∞, ∞	3.000	1.000	0.196	0.793
	v_{novel}	-∞, ∞	3.000	1.000	1.437	0.455
	v_{target}	-∞, ∞	3.000	1.000	0.918	0.642
	z	0, 1	0.500	0.100	0.475	0.052
	$t0$	0, 2	0.300	0.100	0.280	0.116
	$st0$	0, 2	0.100	0.050	0.635	0.219
	p_{gf}	-∞, ∞	0.000	1.000	-2.005	0.507

Supplemental Table 2. Adolescent Brain Cognitive Development Study (ABCD) and Human Connectome Project (HCP) correlations – without covariate adjustment – between efficiency of evidence accumulation (EEA) and whole-network average measures of activation in the cognitive load (2-0) contrast (top panel), as well as correlations of EEA with 0-back and 2-back activation, relative to baseline, for the networks with the strongest associations (bottom panel). 95% confidence intervals, displayed in italics next to each correlation, were estimated using a clustered bootstrapping procedure that accounted for nesting by family and study site. FPN = frontoparietal network; DAN = dorsal attention network; VIS = visual network; SMN = somatomotor network; VAN = ventral attention network; LIM = limbic network; DMN = default mode network.

	**ABCD *r***	**ABCD 95% CI**		**HCP *r***	**HCP 95% CI**	
FPN 2-0	.39	*.36*	*.42*	.50	*.45*	*.55*
DAN 2-0	.25	*.23*	*.28*	.35	*.29*	*.40*
VIS 2-0	-.11	*-.09*	*-.14*	.08	*.02*	*.14*
SMN 2-0	-.31	*-.29*	*-.33*	-.29	*-.22*	*-.35*
VAN 2-0	-.06	*-.03*	*-.10*	.01	*-.06*	*.07*
LIM 2-0	-.19	*-.18*	*-.21*	-.16	*-.10*	*-.23*
DMN 2-0	.01	*-.01*	*.03*	-.05	*-.11*	*.03*
FPN 0	-.29	*-.27*	*-.31*	-.33	*-.27*	*-.38*
FPN 2	.19	*.15*	*.22*	.09	*.02*	*.15*
DAN 0	-.06	*-.03*	*-.09*	-.15	*-.09*	*-.23*
DAN 2	.21	*.18*	*.25*	.10	*.02*	*.17*
SMN 0	.06	*.03*	*.09*	.06	*-.01*	*.13*
SMN 2	-.28	*-.25*	*-.31*	-.12	*-.05*	*-.19*
