# Supplementary material for: Flexible adaptation of task-positive brain networks predicts efficiency of evidence accumulation
Source: Commun Biol. 2024 Jul 2;7:801. doi: 10.1038/s42003-024-06506-w (PMC11220037; doi:10.1038/s42003-024-06506-w)
Supplement: Supplementary file 5 — Reporting Summary [file 42003_2024_6506_MOESM5_ESM.pdf]

nature portfolio

Corresponding author(s): Alexander Weigard

Last updated by author(s): 6/24/2024

Reporting Summary

Nature Portfolio wishes to improve the reproducibility of the work that we publish. This form provides structure for consistency and transparency in reporting. For further information on Nature Portfolio policies, see our Editorial Policies and the Editorial Policy Checklist.

Statistics

For all statistical analyses, confirm that the following items are present in the figure legend, table legend, main text, or Methods section.

n/a	Confirmed	
☐	☒	The exact sample size (*n*) for each experimental group/condition, given as a discrete number and unit of measurement
☐	☒	A statement on whether measurements were taken from distinct samples or whether the same sample was measured repeatedly
☐	☒	The statistical test(s) used AND whether they are one- or two-sided<br>*Only common tests should be described solely by name; describe more complex techniques in the Methods section.*
☐	☒	A description of all covariates tested
☐	☒	A description of any assumptions or corrections, such as tests of normality and adjustment for multiple comparisons
☐	☒	A full description of the statistical parameters including central tendency (e.g. means) or other basic estimates (e.g. regression coefficient) AND variation (e.g. standard deviation) or associated estimates of uncertainty (e.g. confidence intervals)
☐	☒	For null hypothesis testing, the test statistic (e.g. *F*, *t*, *r*) with confidence intervals, effect sizes, degrees of freedom and *P* value noted<br>*Give P values as exact values whenever suitable.*
☐	☒	For Bayesian analysis, information on the choice of priors and Markov chain Monte Carlo settings
☒	☐	For hierarchical and complex designs, identification of the appropriate level for tests and full reporting of outcomes
☐	☒	Estimates of effect sizes (e.g. Cohen's *d*, Pearson's *r*), indicating how they were calculated

Our web collection on statistics for biologists contains articles on many of the points above.

Software and code

Policy information about availability of computer code

Data collection	Neuroimaging and behavioral data used in this study were from existing publicly available data sets (ABCD, HCP). The collection of these data sets is described elsewhere, as detailed below, and relevant information about accessing the data is also available below.
Data analysis	Code for all study analyses can be accessed at: https://osf.io/yte76/.

For manuscripts utilizing custom algorithms or software that are central to the research but not yet described in published literature, software must be made available to editors and reviewers. We strongly encourage code deposition in a community repository (e.g. GitHub). See the Nature Portfolio guidelines for submitting code & software for further information.

Data

Policy information about availability of data

All manuscripts must include a data availability statement. This statement should provide the following information, where applicable:

- Accession codes, unique identifiers, or web links for publicly available datasets
- A description of any restrictions on data availability
- For clinical datasets or third party data, please ensure that the statement adheres to our policy

The ABCD data used in this report came from ABCD release 4.0 (https://nda.nih.gov; DOI 10.15154/1,523,041). ABCD data specific to the current study can be accessed at NDA Study 2297 (DOI 10.15154/wnt8-dq37). HCP data are accessible at: https://db.humanconnectome.org/.

Research involving human participants, their data, or biological material

Policy information about studies with human participants or human data. See also policy information about sex, gender (identity/presentation), and sexual orientation and race, ethnicity and racism.

Reporting on sex and gender	Sex, as a biological attribute, was included as a covariate in all study analyses. Sex was self-reported in both the ABCD and HCP samples. As detailed in the manuscript and supplemental materials, sensitivity analyses indicated that inclusion of sex as a covariate had little effect on the substantive pattern of results reported in this study.
Reporting on race, ethnicity, or other socially relevant groupings	Individuals' self-reported race (e.g., White, Black, Asian) and Hispanic ethnicity were both used as covariates in all study analyses. As detailed in the manuscript and in supplemental materials, sensitivity analyses indicated that inclusion of race and ethnicity as covariates had little effect on the substantive pattern of results reported in this study.
Population characteristics	See below.
Recruitment	Detailed descriptions of the recruitment methods for the ABCD sample and HCP sample are available in Garavan et al. (2018, Developmental Cognitive Neuroscience) and Van Essen et al. (2013, Neuroimage), respectively. Briefly, the ABCD Study recruited a diverse sample of over 11,000 9-10 year old youth across 21 consortium sites in the United States. The recruitment strategy was designed to obtain a sample as close to the demographic distribution of the United States (e.g., with regard to race, ethnicity, income, and educational attainment) as possible. The HCP sample was recruited from a population of adult twins and their non-twin siblings, ages 22-35 years.
Ethics oversight	All procedures for the HCP study were approved by the Washington University Institutional Review Board (IRB). The majority of ABCD sites rely on a central IRB at the University of California, San Diego for ethical oversight, although a few rely on local IRBs at their specific institution for ethical oversight. Study procedures for all ABCD sites were also approved by each site's local IRB prior to the beginning of the study.

Note that full information on the approval of the study protocol must also be provided in the manuscript.

Field-specific reporting

Please select the one below that is the best fit for your research. If you are not sure, read the appropriate sections before making your selection.

☐ Life sciences ☒ Behavioural & social sciences ☐ Ecological, evolutionary & environmental sciences

For a reference copy of the document with all sections, see nature.com/documents/nr-reporting-summary-flat.pdf

Behavioural & social sciences study design

All studies must disclose on these points even when the disclosure is negative.

Study description	Quantitative study of associations between multivariate brain imaging data and parameters from a computational model that describes cognitive task performance.
Research sample	The project uses archived open-access data from two large samples, the ABCD Study and HCP.
Sampling strategy	Detailed descriptions of the sampling strategies used for the ABCD sample and HCP sample are available in Garavan et al. (2018, Developmental Cognitive Neuroscience) and Van Essen et al. (2013, Neuroimage), respectively.
Data collection	In both open-access samples investigated in this study, magnetic resonance imaging (MRI) measures were collected using 3-Tesla MRI machines and trial-level data from the n-back task were recorded simultaneously using a computer program. Data on participant demographic covariates was collected from participants' responses on questionnaire measures. All types of data were accessed by our study team from repositories for each of the open-access samples.
Timing	The ABCD baseline data used in the current study were collected between January 2017 and September 2018. The HCP project began in 2010 and collected data over the subsequent 6 years.
Data exclusions	Individual neuroimaging runs were excluded if they failed visual inspection or had less than 4 minutes of data remaining after images with >0.9mm framewise displacement were censored from the imaging time series. Individual subjects were excluded if they had less than two included neuroimaging runs, did not have complete behavioral task data, or showed clear evidence of disengagement from either the 0-back or 2-back tasks (accuracy <55%, omissions/non-responses >25%). These criteria led to the inclusion of 4,315 out of the original 11,875 participants in the ABCD sample and 883 of the original 1,206 participants in the HCP sample.
Non-participation	Not applicable, as data were already collected.
Randomization	This was an observational study, and randomization was therefore not used.

Reporting for specific materials, systems and methods

We require information from authors about some types of materials, experimental systems and methods used in many studies. Here, indicate whether each material, system or method listed is relevant to your study. If you are not sure if a list item applies to your research, read the appropriate section before selecting a response.

Materials & experimental systems

n/a	Involved in the study
☒	☐ Antibodies
☒	☐ Eukaryotic cell lines
☒	☐ Palaeontology and archaeology
☒	☐ Animals and other organisms
☒	☐ Clinical data
☒	☐ Dual use research of concern
☒	☐ Plants

Methods

n/a	Involved in the study
☒	☐ ChIP-seq
☒	☐ Flow cytometry
☐	☐ MRI-based neuroimaging

Magnetic resonance imaging

Experimental design

Design type: Blocked design

Design specifications: These specifications are detailed for the ABCD and HCP samples in Casey et al. (2018, Developmental Cognitive Neuroscience) and Van Essen et al. (2013, Neuroimage), respectively.

Behavioral performance measures: Response times and accuracy rates from the 0-back and 2-back tasks were analyzed with a computational model, the diffusion decision model (DDM), to obtain parametric estimates of mechanisms underlying task performance.

Acquisition

Imaging type(s): Functional

Field strength: 3T

Sequence & imaging parameters: These parameters are detailed for the ABCD and HCP samples in Casey et al. (2018, Developmental Cognitive Neuroscience) and Van Essen et al. (2013, Neuroimage), respectively.

Area of acquisition: Whole brain

Diffusion MRI: ☐ Used ☒ Not used

Preprocessing

Preprocessing software: fMRIPrep version 1.5.0

Normalization: Cortical surface data were transformed to CIFTI space with 91,282 grayordinates.

Normalization template: CIFTI surface space.

Noise and artifact removal: Images were high pass filtered at 0.005 Hz. Nuisance covariates in the first level models consisted of 24 motion correction parameters (3 rotation, 3 translation, first derivatives of each, and quadratics of original and derivatives), top 5 principal components of signal from white matter, top 5 principal components of signal from cerebrospinal fluid, and individual regressors for each TR that exceeded a 0.9mm framewise displacement.

Volume censoring: TRs with >0.9mm framewise displacement were censored.

Statistical modeling & inference

Model type and settings: Task conditions modeled included 0-back and 2-back for each category of stimuli (HCP: faces, places, body parts, tools; ABCD: happy faces, neutral faces, fearful faces, places) along with the nuisance covariates described above. Linear contrasts were constructed for contrasts of interest: 0-back, 2-back, 2-back vs 0-back. Brain-wide parameter estimates from these first-level models were then entered into multivariate predictive models to predict parameters estimated from task behavior and used to compute multivariate summary measures of brain network dynamics, as described in Methods,

Effect(s) tested

Associations between multivariate measures of brain activation and parameter estimates from the diffusion decision model of behavior.

Specify type of analysis: ☒ Whole brain ☐ ROI-based ☐ Both

Statistic type for inference

(See Eklund et al. 2016)

Cross-validation in unseen data was used to evaluate the accuracy of multivariate predictive models and correlation tests were used to estimate associations between multivariate summary measures and behavioral performance measures. For both types of associations, Pearson's r value was used to quantify effect size. Confidence intervals (CIs) for r values were obtained using a clustered bootstrapping method that accounted for the nested structure of the data in both samples (HCP famlieis; ABCD families and sites). All analyses accounted for demographic covariates and participant in-scanner motion.

Correction

As this study does not use a mass univariate approach, spatial corrections used in mass univariate neuroimaing analyses are not relevant.

Models & analysis

n/a	Involved in the study
☒	☐ Functional and/or effective connectivity
☒	☐ Graph analysis
☐	☒ Multivariate modeling or predictive analysis

Multivariate modeling and predictive analysis

Subject level contrast images for 2-back vs 0-back were used in a cross-validated principal components regression (PCR) predictive model. In brief, this method performs dimensionality reduction on input data, fits a regression model on the resulting components, and applies this model out of sample in a 10-fold (HCP) or leave-one-site-out (ABCD) cross-validation framework. Nuisance covariates (age, age squared, sex, race/ethnicity, framewise displacement estimate of motion, framewise displacement squared) are handled by calculating a cross-validated form of partial correlation. In each training fold after the PCA is conducted to reduce the data, K components are retained, with the optimal value for K being estimated with a nested 5-fold cross-validation within just the training data. Both the component expressions as well as the outcome variable are regressed against nuisance variables. The betas estimated from this model are used to residualize both the training and test data. Then a linear model is fit on the training data to predict the residualized outcome with the residualized expressions. This model is subsequently applied to the test data to obtain a predicted value, which can then be correlated with the residualized outcome to obtain an out-of-sample partial correlation estimate. This is repeated for each fold and the per-fold correlations are summarized by averaging and by estimating a 95% confidence interval (CI) for the average values.
